# A new algorithm for hip fracture surgery: Reoperation rate reduced from 18% to 12% in 2,000 consecutive patients followed for 1 year

**DOI:** 10.3109/17453674.2011.652887

**Published:** 2012-02-08

**Authors:** Henrik Palm, Michael Krasheninnikoff, Kim Holck, Tom Lemser, Nicolai Bang Foss, Steffen Jacobsen, Henrik Kehlet, Peter Gebuhr

**Affiliations:** ^1^Departments of Orthopaedics; ^2^Anaesthesiology, Hvidovre University Hospital; ^3^Section of Surgical Pathophysiology, the Juliane Marie Centre, Rigshospitalet, Copenhagen, Denmark

## Abstract

**Background and purpose:**

Treatment of hip fracture patients is controversial. We implemented a new operative and supervision algorithm (the Hvidovre algorithm) for surgical treatment of all hip fractures, primarily based on own previously published results.

**Methods:**

2,000 consecutive patients over 50 years of age who were admitted and operated on because of a hip fracture were prospectively included. 1,000 of these patients were included after implementation of the algorithm. Demographic parameters, hospital treatment, and reoperations within the first postoperative year were assessed from patient records.

**Results:**

931 of 1,000 operative procedures were performed according to the algorithm, as compared to only 726 of 1,000 prior to its introduction (p < 0.001). After implementation of the algorithm, junior registrars still performed half of the operations, but unsupervised procedures declined from 192 of 1,000 to 105 of 1,000 (p < 0.001). The rate of reoperations declined from 18% to 12% (p < 0.001 in a multiple Cox regression analysis), with a decline of 24% to 18% for intracapsular fractures and a decline of 13% to 7% for extracapsular fractures. The proportion of bed-days caused by reoperations was reduced from 24% of total hospitalization before the algorithm was introduced to 18% after it was introduced.

**Interpretation:**

It is possible to implement an algorithm for treatment of all hip fracture patients in a large teaching hospital. In our case, the Hvidovre algorithm both raised the rate of supervision and reduced the rate of reoperations. The reduced reoperation rate saved many hospital bed-days.

The surgical treatment of hip fracture patients is controversial, with high reoperation rates and long hospitalization time (Foss et al. 2007, Palm et al. 2006). In the last decades, some evidence has been put forward for more optimized treatment and general guidelines have appeared (Kyle et al. 1995, Parker and Gurusamy 2005, Palm et al. 2006, Danish Orthopedic Society 2008). However, in everyday clinical practice, the exact choice of implant often remains controversial, and here an easily used surgical algorithm for all hip fracture patients is warranted. To our knowledge, no such algorithm has ever been presented.

Based on a review of the literature and own previously published studies of predictors for reoperation (Palm et al. 2006, 2007a, 2009, 2011), we developed an evidence-based algorithm for the surgical treatment of all hip fracture patients—solely based on fracture classification and patient age (Figure). We included demands for supervision, as unsupervised junior registrars have been shown to be an independent risk factor for reoperation (Palm et al. 2007b).

**Figure:**
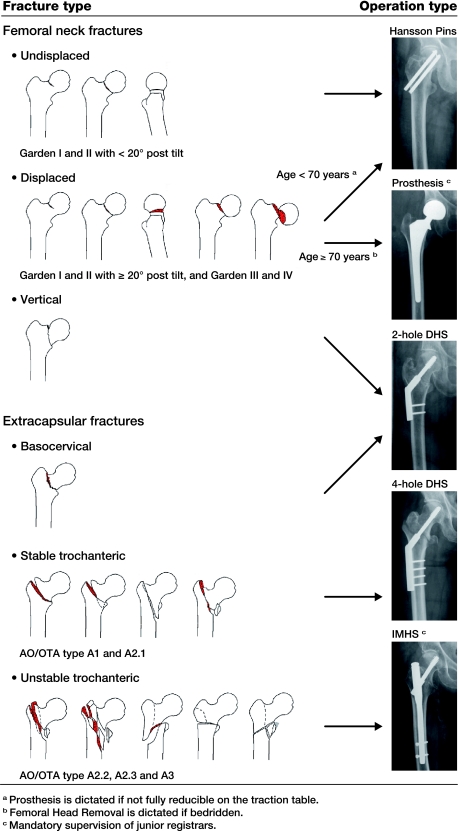
The algorithm for hip fracture surgery.

We investigated whether such an algorithm could be implemented in everyday clinical practice at a large teaching hospital and whether it could reduce the need for reoperations.

## Patients and methods

2,000 consecutive patients aged over 50 years were admitted to Hvidovre Hospital between September 2002 and July 2009 after having sustained a hip fracture, and they were prospectively included in a database. In March 2006, when 1,000 patients had been included, the algorithm was implemented prospectively for the next 1,000 patients.

The algorithm, as a 80 × 120 cm chart, was placed in the departmental conference room, in the hallway of the operating theater, and in the (separate) hip fracture ward. It was also distributed as a pocket-size version, and plenum education for surgeons was held twice a year. Prior to implementation of the algorithm, the surgeon on duty chose the implant independently. After implementation, the choice of implant had to follow the algorithm, solely based on fracture classification on preoperative radiographs (anterior-posterior and lateral), patient age, and in rare cases whether the patient was bedridden before the fracture.

Patients were divided into 7 treatment groups. (1) Undisplaced femoral neck fractures (Garden type I–II with less than 20° posterior tilt) (Palm et al. 2009) were operated with 2 parallel implants (Hansson Pin System; Swemac Orthopaedics AB, Linköbing, Sweden). Displaced femoral neck fractures (Garden type I–II with more than 20° posterior tilt and Garden type III–IV) (Palm et al. 2009) were treated based on patient age. (2) Patients less than 70 years old were operated with the 2 parallel implants—except if the fracture could not be anatomically reduced on the fracture table, in which case a prosthesis was inserted (Biomet Fracture Stem; Scan Bi Polar Head, Biomet, Warsaw, IN). (3) Patients who were 70 years and older were always given a prosthesis, but to reduce the risk of infection and dislocation, the femoral head was simply removed in the rare pre-fracture bedridden patients. (4) Vertical femoral neck fractures (Parker 2009) and (5) basocervical fractures (Mallick and Parker 2004) were given a sliding hip screw (HipLOC Dynamic Compression Screw mounted on a 2-hole 135° side-plate; Biomet, Warsaw, IN). (6) Stable trochanteric fractures (AO/OTA type 31A1 and A2.1) (Orthopaedic Trauma Association 2007) were operated with the same sliding hip screw, but mounted on a 4-hole 135° side-plate. (7) Unstable trochanteric fractures (AO/OTA type 31A2.2-A2.3 and A3) (Orthopaedic Trauma Association 2007) were operated with an antegrade intramedullary nail (130° IMHS; Smith and Nephew, Memphis, TN). Supervision of junior registrars (Palm et al. 2007) was mandatory for prostheses and intramedullary nails, while an individual license could be obtained by junior registrars for the other procedures.

Cognitive function (Qureshi et al. 1974), the ASA physical grading score (American Society of Anaesthesiologists 1963) and the New Mobility score (NMS) (Parker and Palmer 1993) were determined for each patient. All patients were managed with the department's specialized hip-fracture multimodal fast-track protocol, and operated during daytime with epidural analgesia (Foss et al. 2005). Preoperatively, a single dose of 1.5 g of cephalosporin was administered intravenously. Postoperatively, low-molecular-weight heparin was administered until the patient was fully mobilized, but for a minimum of 5 days. Full weight bearing from the day of the surgery was encouraged in an intensive physiotherapy program. The rate of reoperation within the first year was registered from patient records and cross-checked with the Copenhagen radiographic database. The department's guideline for need of a reoperation was unchanged during the study period and reoperations for all causes were registered as outcome parameter.

The study was part of the hip fracture project at Hvidovre University Hospital, Copenhagen, Denmark. It was approved by the Danish Data Protection Agency, and the Copenhagen Ethics Committee concluded that written patient consent was not required.

### Statistics

The number of patients required was estimated from a power analysis with a power of 80 and a significance level of 0.05, with the hypothesis of a 30% reduced reoperation rate for both intracapsular and extracapsular fractures. Differences in demographic and clinical parameters were analyzed using chi-square tests for dichotomized data, Mann-Whitney tests for patient age, and Kaplan-Meier test for patient survival. The overall reoperation rate was compared by multiple Cox regression analysis on time to reoperation. Patients were censored after 1 year or, if dead, within a year, and analysis was adjusted for age, sex, ASA, NMS, and cognitive function. Subgroup differences were analyzed with univariate Cox regression analysis. The level of significance was set at p < 0.05. All calculations were performed with SPSS statistical software version 16.0.

## Results

Age, sex distribution, and cognitive function were similar in the 2 groups, whereas high ASA score and high NMS were commoner in patients who were treated before the algorithm (Table 1). Except for dislocation, all major reasons for reoperation were reduced, and the overall number of reoperations declined from 18% to 12%. From a multiple Cox regression analysis (Table 2), use of the algorithm statistically significantly reduced the risk of reoperation, as did higher patient age.

**Table 1.** Data for the 2,000 patients who were operated either before or after implementation of the algorithm

	Before algorithm		After algorithm		
	n	%	n	%	p-value
No. of patients	1,000		1,000		
Age (years) **^a^**	83 (76–89)		83 (74–89)		0.7
Female gender	749		742		0.7
ASA score III–IV	517		291		< 0.001
Prefracture NMS 0–5	530		434		< 0.001
Low cognitive function	297		287		0.6
Dead within 1 year **^b^**	307		306		0.6
Reoperations within 1 year	180	100	124	100	< 0.001
Hematoma/Bleeding	3	2	3	2	
Infection	26	15	11	9	
Dislocation of prosthesis	20	11	21	17	
Loosening of prosthesis	6	3	2	2	
Periprosthetic fracture	25	14	12	10	
Avascular necrosis of femoral head	8	4	5	4	
Nonunion	51	28	37	29	
Cutout of implant into hip joint	23	13	15	12	
Fracture around the implant	8	5	9	7	
Distal locking screw position	2	1	2	2	
Subsequent osteoarthritis	2	1	1	1	
Implant removal due to pain/discomfort	6	3	6	5	

Values are presented as number of patients and were analyzed using the chi-square test, except for:

**^a^** Age, presented as median (interquartile range) and analyzed using Mann-Whitney test.

**^b^** Dead within 1-year, analyzed using Kaplan-Meier test. ASA: American Society of Anaesthesiologists; NMS: New Mobility score.

**Table 2.** Relationship between reoperation within 1 year postoperatively and patient characteristics in the 2,000 patients who were operated before or after implementation of the algorithm

		Multiple Cox regression analysis		
	n (%)	HR	(95% CI)	p-value
Age (years) **^a^**	83 (75–89)	1.0	(1.0–1.0)	0.002
Female gender	1,491 (75)	1.2	(0.9–1.5)	0.3
ASA score III–IV	808 (40)	1.2	(0.9–1.5)	0.2
Prefracture NMS 0–5	964 (48)	0.8	(0.6–1.0)	0.08
Low cognitive function	584 (29)	0.8	(0.6–1.1)	0.2
Surgical algorithm	1,000 (50)	0.7	(0.5–0.8)	< 0.001

**^a^** Age presented as median (interquartile range). HR: hazard ratio; ASA: American Society of Anaesthesiologists; NMS: New Mobility score.

A decline in reoperations was seen for both the intracapsular and extracapsular fractures (Table 3). A decline in reoperations was especially seen for displaced femoral neck fractures in patients over 70 years of age and for unstable trochanteric fractures. 726 of the 1,000 patients admitted before the algorithm was implemented were operated in a similar way to that suggested by the principles used in the algorithm. After implementation, the compliance with the recommendations in the algorithm was 931 of 1,000 operative procedures (p < 0.001).

**Table 3.** Fracture type, operation type, and rate of reoperation for the 2,000 patients who were operated before or after implementation of the algorithm

	Before algorithm		After algorithm		
		Reop.		Reop.	
Fracture type		rate		rate	
Type of operation	n	(%)	n	(%)	p-value
*Femoral neck fractures*	467	24	482	18	0.02
Undisplaced	88	17	79	13	0.3
Parallel implants **^a^**	75		74		
Prosthesis	9		5		
Other	4		–		
Displaced (Age < 70 years)	37	51	61	27	0.5
Parallel implants **^a^**	25		37		
Prosthesis **^b^**	8		23		
Other	4		1		
Displaced (Age ≥ 70 years)	329	22	328	13	0.005
Prosthesis **^a^**	288		316		
Femoral head removal **^c^**	2		3		
Parallel implants	38		7		
Other	1		2		
Vertical	13	39	14	43	0.7
DHS (2 holes) **^a^**	9		11		
DHS (other types)	1		1		
Parallel implants	3		2		
*Extracapsular fractures*	533	13	518	7	0.002
Basocervical	22	9	21	14	0.7
DHS (2 holes) **^a^**	13		20		
DHS (other types)	7		–		
Parallel implants	–		1		
Prosthesis	2		–		
Stable trochanteric	211	7	144	4	0.3
DHS (4 holes) **^a^**	204		136		
DHS (other types)	4		–		
IMHS	3		8		
Unstable trochanteric	300	17	353	8	< 0.001
IMHS **^a^**	112		337		
DHS (4 holes)	156		13		
DHS (other types)	32		–		
Other implants	–		3		
All hip fractures	1,000	18	1,000	12	< 0.001
Level of surgeon					
Unsupervised junior registrar	192	20	105	11	0.03
Supervised junior registrar	287	16	398	10	0.02
Senior registrar or consultant	521	18	497	15	0.08

**^a^** Operation type dictated by the algorithm.

**^b^** Prosthesis is dictated if not fully reducible on the traction table.

**^c^** Femoral head removal is dictated if bedridden. Rates of reoperation were analyzed using univariate Cox regression analysis.

106 different surgeons took part in the 2,000 surgical procedures. 41 of them operated patients before implementation of the algorithm, 40 after implementation, and 25 both before and after implementation. Unsupervised procedures declined from 192 procedures before implementation to 105 after implementation (p < 0.001), but junior registrars still performed half of the operations. A reduction in reoperation rate was seen both for the junior registrar procedures with supervision (16% to 10%, p = 0.02) and without supervision (20% to 11%, p = 0.03).

The total number of orthopedic bed-days—including re-admissions due to reoperations within 1 year—was 35,284; that is, 20,031 bed-days before implementation of the algorithm and 15,253 bed-days after implementation. Assuming that the patients with reoperations would otherwise have had the same average length of stay as the remaining patients (15.2 days before and 12.5 days after the algorithm) (Foss et al. 2007), the consumed extra bed-days caused by reoperations was reduced from 24% ((20,031 – 15,200) / 20,031) before implementation of the algorithm to 18% ((15,253 – 12,500) / 15,253) after implementation—corresponding to an estimated reduction of 900 bed-days.

## Discussion

We found that an algorithm for the heterogeneous hip fracture patient population can be implemented and used by different surgeons in everyday clinical practice in a large teaching hospital. The overall reoperation rate was thereby reduced from a higher than average level to a slightly lower level than in the literature (Parker and Gurusamy 2005). The patients in our study were, however, unselected and consecutive, and therefore also included very fragile patients who would not normally be included in trials requiring written patient consent. The primary hospitalization was reduced from an average of 15 days to 12 days, but apart from the new algorithm, this might reflect a healthier patient population, improved perioperative treatment, and changed possibilities for discharge in the community. We therefore chose the conservative calculation described, but still found a drastic reduction in the rate of bed-days spent on reoperations. This improvement might be partly explained by an increased amount of attention in general during the early phase of implementation, but this could hardly be the reason during the whole study period.

93% of procedures followed the directions of the algorithm in the strictest sense, and a higher level of fulfillment was probably not achievable due to individual patient biology. As with classification systems, a higher degree of detail would only make the algorithm less useful in everyday practice. Dedicated and highly specialized surgical teams have given improved results (Parker et al. 1994), but because hip fractures represent a large proportion of trauma cases in most orthopedic departments, logistics often make it impossible to reserve these procedures for a few specific consultants. In the common orthopedic setup, hip fractures also make up an important part of junior registrars' education, but the higher risk of failure with unsupervised procedures should be remembered (Palm et al. 2007b). With many different surgeons performing hip fractures at our institution, we therefore chose to incorporate strict demands for supervision in the algorithm. This was well accepted in everyday clinical practice, as the junior registrars experienced better backup and the older surgeons expressed more awareness of their younger colleagues' surgical level. Junior registrars still performed half of all procedures, but the number of unsupervised procedures was reduced and the overall reduction in reoperations was mainly seen with the junior procedures (Table 3). It is, however, difficult to determine whether the improved result was due to the increased rate of supervision or to a better choice of implant.

In contrast to previous general guidelines (Kyle et al. 1995, Parker and Gurusamy 2005, Palm et al. 2006, Danish Orthopedic Society 2008), we chose to use an algorithm with strict recommendations for all hip fracture patients, also in areas not supported by level-1 evidence. This is debatable, but the lower number of reoperations supports the value of our overall recommendations. Improvement was mainly seen in the 2 patient groups with most changes in surgical technique. The use of prostheses was increased and parallel implants decreased in displaced femoral neck fractures in patients above 70 years of age, and treatment of patients with unstable trochanteric fractures shifted from dynamic hip screws to intramedullary nails. The reduced reoperation rates support the idea that parallel implants and dynamic hip screws appear to be insufficient in these patient groups (Bhandari et al. 2003, Rogmark et al. 2006, Palm et al. 2007a, 2009, 2011)—although future level-1 evidence studies including outcome parameters such as pain, mobility, and activities of daily living, not available in this cohort study, should be taken into account.

The algorithm also highlighted some remaining problems, one being the continued unacceptably high failure rate in younger patients treated with parallel implants for femoral neck fractures. The age for receiving prostheses could be lowered, but this would probably increase the number of late revisions, as more patients would outlive their prosthesis. Parallel implants may also fail if there is lack of varus support by the calcar (Alho et al. 1991), as seen in basocervical fractures (Mallick and Parker 2004) and in the debatable subgroup of vertical fractures (Parker 2009). In spite of the decision to use a fixed-angle device, the high failure rate persisted. A treatment algorithm should not be static. So far, we have reached an overall reoperation rate of 12% in unselected hip fracture patients, but with the appearance of new treatment evidence this algorithm will be developed further.
